# Exploring the Potential Role of ADAM 17 and ADAM 22 in the Etiology of Autism Spectrum Disorders

**DOI:** 10.3390/brainsci13060972

**Published:** 2023-06-20

**Authors:** Sarah H. Al-Mazidi, Afaf El-Ansary, Amani Abualnaja, Abdullah AlZarroug, Turki Alharbi, Laila Y. Al-Ayadhi

**Affiliations:** 1 Department of Physiology, Faculty of Medicine, Imam Mohammed Ibn Saud Islamic University, Riyadh 11432, Saudi Arabia; s.almazeedi@gmail.com (S.H.A.-M.); amani.abualnjaa8@gmail.com (A.A.); afmalzarroug@sm.imamu.edu.sa (A.A.); turki.7123@gmail.com (T.A.); 2 Autism Center, Lotus Holistic Alternative Medical Center, Abu Dhabi 110281, United Arab Emirates; 3 Autism Research and Treatment Centre, King Saud University, Riyadh 11461, Saudi Arabia; ayadh2@gmail.com; 4 Department of Physiology, Faculty of Medicine, King Saud University, Riyadh 11461, Saudi Arabia

**Keywords:** metalloproteases, ADAM17, ADAM22, neuroinflammation, glutamate excitotoxicity, gut–brain axis

## Abstract

Background: Autism spectrum disorder (ASD) encompasses a group of disorders characterized by difficulties with social interaction and repetitive behavior. The condition is supposed to originate from early shifts in brain development, while the underlying processes are unknown. Moreover, a considerable number of patients with ASD experience digestive difficulties. Metalloproteases (ADAMs) are a class of enzymes capable of cleaving membrane-bound proteins. Members of this family, ADAM17 and ADAM22, have the ability to cleave proteins like the pro-inflammatory cytokine TNF-ά and glutamate synaptic molecules, which are both engaged in neuro-inflammation and glutamate excitotoxicity as crucial etiological mechanisms in ASD. ADAM17 and ADAM22 may also have a role in ASD microbiota–gut–brain axis connections by regulating immunological and inflammatory responses in the intestinal tract. Subjects and Methods: Using ELISA kits, the plasma levels of ADAM17 and ADAM22 were compared in 40 children with ASD and 40 typically developing children. All of the autistic participants’ childhood autism rating scores (CARS), social responsiveness scales (SRS), and short sensory profiles (SSP) were evaluated as indicators of ASD severity. Results: Our results showed that plasma levels of ADAM17 were significantly lower in ASD children than in control children, while ADAM22 demonstrated non-significantly lower levels. Our data also indicate that while ADAM17 correlates significantly with age, ADAM22 correlates significantly with CARS as a marker of ASD severity. Conclusions: Our interpreted data showed that alteration in ADAM17 and ADAM22 might be associated with glutamate excitotoxicity, neuroinflammation, and altered gut microbiota as etiological mechanisms of ASD and could be an indicator of the severity of the disorder.

## 1. Introduction

Autism spectrum disorder (ASD) encompasses a group of neurodevelopmental diseases typically identified in infancy but which may last throughout adulthood. It is mainly indicated by a lack of social engagement and communication as well as by the existence of stereotyped behaviors [1]. 

ASD is a collection of neurodevelopmental disorders characterized by social and behavioral abnormalities, difficulty in verbal and nonverbal communication, and confined, repetitive behaviors and interests. The World Health Organization estimates that the global prevalence of ASD in 2022 is almost one in every 36 individuals [2].

In addition to synapse-related genes being downregulated, microglia and immune-related genes were upregulated in autistic patients’ brains [3,4]. The pathogenic pathway also includes linkages between astrocytes, microglial activation, neuroinflammation produced by gut microbiota, and immunological dysregulation in ASD patients [5,6,7]. Additionally, there is well-established evidence that ASD patients have altered glutamate circuits and GABAergic circuits, as shown by elevated excitatory synapses and spine densities, markedly decreased glutamic acid decarboxylase levels, and altered GABAA and GABAB receptors in the postmortem brains of autistic patients [8].

The disintegrin and metalloprotease (ADAMs) family of proteins are type I transmembrane and secreted metalloendopeptidases that mediate the cleavage of membrane protein extracellular domains (ectodomains). ADAMs regulate a variety of proteins, including growth factors, cytokines, receptors, and adhesion molecules, all of which play critical roles in a variety of biological processes such as cell migration, cell adhesion, proteolysis, and signal transmission [9]. 

ADAM 17 and ADAM 22 enzymes are members of the family of metalloproteases (ADAMs) that can degrade membrane-bound proteins. ADAM17 and ADAM22 cleave essential protein substrates that govern neuronal networks and immunological responses, respectively, such as the neuro-inflammatory cytokine TNF-α and glutamate synaptic molecules, all of which are involved or disturbed in ASD [10].

Higher brain operations like learning and memory rely on finely regulated synaptic transmission and plasticity [11]. PSD-95 is a scaffolding protein that regulates the synaptic localization of many receptors, including AMPA and NMDA glutamate receptors, channels, and signaling proteins, and thus is required for proper excitatory synaptic transmission and postsynaptic glutamate signaling. LGI1, an epilepsy-related secreted protein, and its receptor ADAM22 are members of the PSD-95-containing synaptic protein complex in the brain [12]. According to current research, the LGI1-ADAM22 complex plays a key role in AMPA and NMDA receptor-mediated synaptic transmission via regulating membrane-associated guanylate kinases (MAGUKs) [13]. Interestingly, PSD-95 cannot boost AMPA receptor-mediated synaptic transmission without interacting with LGI1-ADAM22. It is well-accepted that AMPA receptors are less functional in ASD individuals [12,13].

The gut–brain axis denotes the bidirectional pathway between the gut and the brain [14]. Alterations in this pathway in ASD patients could lead to the increased permeability of both the intestinal and brain barriers. A leaky gut and disrupted blood–brain barrier, as a well-documented phenotype in ASD, will increase the permeability of barriers, allowing cytokines and bacterial metabolites to enter the brain directly, leading to neuroinflammation and neuronal dysfunction [15,16]. It is well accepted that gut microbiota can influence ADAMs proteases by generating bacterial metabolites and that these proteases can affect the composition of the gut microbiome. However, these linkages are as yet unknown and more research is required [17].

In an attempt to link neuroinflammation, glutamate excitotoxicity, and impaired gut microbiota as etiological pathways in ASD that are regulated by ADAM 17 and ADAM 22 respectively, new evidence has revealed that TNF-ά has been shown to increase excitatory synaptic strength via AMPA receptor exocytosis and reduce inhibitory synaptic strength via GABA (A) receptor endocytosis, shifting the E/I balance towards excitation [18,19].

El-Ansary [20] proposed that inactivating NMDA and AMPA glutamate receptors while activating GABA (A) receptors could aid in reversing glutamate excitotoxicity, hence supporting glutamate-centered ASD hypotheses [21,22]. Ray et al. [23] discovered a considerably higher soluble ADAM17 level in ASD patients’ brain tissue, although its involvement in ASD pathophysiology remains unknown.

Because there is no particular biomarker for ASDs, they are frequently not properly diagnosed. The discovery of possible targets for the development of diagnostic and/or therapeutic procedures must be given top priority in ASD care in the future. Numerous proteases are connected to both the regulatory mechanisms of ASD etiopathology and therapy as well as the physiological processes that underlie these disorders. 

In genetic animal models of ASD, the AMPA receptor positive allosteric modulator increased social interaction [24]. These findings suggest that a novel approach to treating the symptoms of ASD might involve enhancing excitatory synaptic transmission through the AMPA receptor. Based on this, proteases may be thought of as novel targets to gain a greater understanding of the inflammatory, excitotoxic, and immunological molecular processes linked to the etiology of ASD. 

**Figure 1:**
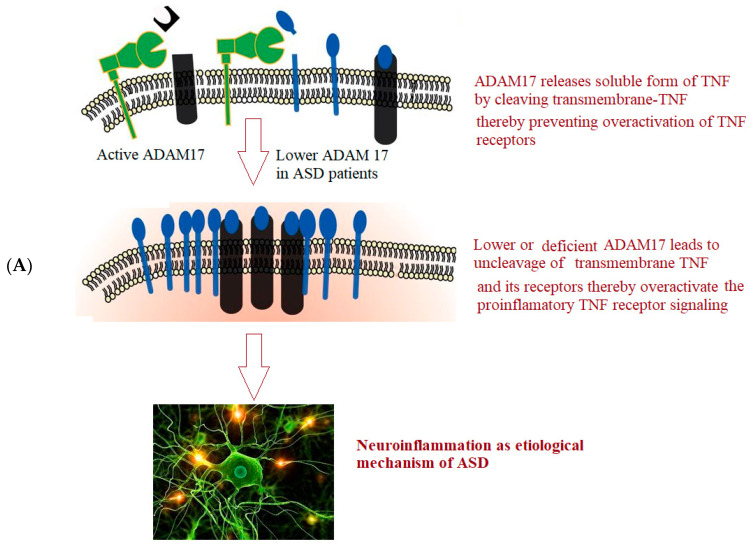

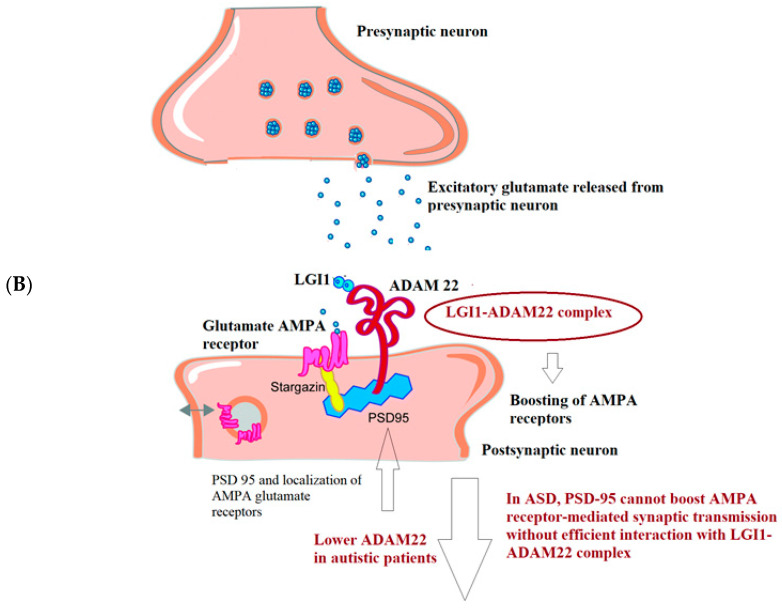
Role of ADAM17 (**A**) and ADAM22 (**B**) in neuroinflammation and glutamate signaling in ASD respectively.

This information tempered our interest in measuring ADAM 17 and ADAM 22 in the plasma of individuals with ASD to emphasize the role of both proteases in neuroinflammation and unbalanced E/I neurotransmission as two known etiological mechanisms of ASD previously reported in the same cohort of participants [24]. Figure 1 depicts the hypothesized roles of ADAM17 (A) and ADAM22 (B) in neuroinflammation and glutamate signaling in ASD.

## 2. Methods

The Autism Research and Treatment Center, Faculty of Medicine, King Saud University, and King Khalid University Hospital were the sites of this study. The King Saud University Faculty of Medicine’s Institutional Review Board authorized the study protocol following the most recent Declaration of Helsinki (Ref. No. 22/0122/IRB). Before the assessment, all participants’ parents provided informed consent. The Diagnostic and Statistical Manual of Mental Disorders, Fourth Edition, was used to diagnose ASD (DSM-IV).

### 2.1. Participants

This study included 80 children (40 ASD children and 40 healthy children). ASD male and female children aged 3 to 12 (mean age of 6.43 ± 2.22) who met the inclusion criteria for this study were recruited from the Autism Research and Treatment Center, Faculty of Medicine, King Saud University. The control group consisted of 40 age-matched healthy male and female children who visited King Khalid University Hospital’s pediatric clinic for normal check-ups. The study excluded anyone with an infectious condition or a mental problem. Patients who were associated with neurological disease (such as palsy and tuberous sclerosis), metabolic disorders (e.g., phenyl ketonuria, diabetes), and autoimmune disease were excluded from the study, as metabolic diseases and autoimmunity may influence the results of plasma ADAM 17 and ADAM 22 levels. The control group consisted of 40 healthy male and female children who were matched for age and sex. They had no family relations to the ASD children, and their ages ranged from 3 to 12 (5.83 ± 3.22 years). Additionally, they did not have any clinical signs of immunological, diabetic, chronic, or neuropsychiatric illnesses. 

### 2.2. Behavioral Assessment

#### Childhood Autism Rating Score (CARS)

CARS is commonly used to screen for autism [25]. It is a 15-domain observational scale intended to differentiate children with ASD from other types of effects. The CARS evaluates the child on a scale of 1 (normal) to 4 (severe abnormality); higher scores indicate a greater impairment. Items relating to people, emotional response, imitation, body use, object use, listening response, fear or nervousness, verbal communication, nonverbal communication, activity level, level and consistency of intellectual response, adaptation to change, visual response, taste, smell, and touch response, and general impressions are among the domains. Overall scores vary from 15 to 60; a score of less than 30 suggests a non-autistic range; a score of 30 to 36.5 indicates mild to moderate autism; and a score of 37 to 60 points indicates severe autism.

### 2.3. Social Responsiveness Scale

The SRS is a 65-item scale for assessing the severity of autistic symptoms [26]. It is a quantitative questionnaire that parents and teachers must answer in 15–20 min based on the child’s observed behavior over the previous six months. It features a standard 4-point scale ranging from “0” (false) to “3” (almost always true). This measure is divided into five subscales: social awareness, cognition, communication, motivation, and autistic mannerisms. An SRS score of 60 to 75 indicates a modest to moderate level of social impairment. A score of 76 or above, on the other hand, is considered as serious social impairment.

### 2.4. Short Sensory Profile (SSP)

The SSP is a 38-item questionnaire used to score a variety of sensory deficits [27]. It is completed by the child’s caretaker. Each SSP item is scored on a 5-point Likert scale (1 being “always” and five being “never”). The questionnaire has seven subscales: tactile sensitivity (7 things), taste/smell sensitivity (4 items), movement sensitivity (3 items), desiring sensation (7 items), auditory filtering (6 items), low-energy levels (6 items), and visual/auditory sensitivity (7 items/5 items). Lower scores imply a stronger link with ASD. Scores vary from 38 to 190; lower values indicate higher degrees of sensory behavior; the full sensory response is classified as follows: Scores between 142 and 152 suggest modest to moderate performance (most significant frequency of sensory complaints), whereas scores between 153 and 190 show no symptoms.

### 2.5. Measurement of Plasma ADAM17 and ADAM22

A Collection of plasma samples:

After overnight fasting, blood was collected using EDTA or heparin as an anticoagulant. Samples were then centrifuged for 15 min at 1000× *g* at 2–8 °C within 30 min of collection. The samples were stored at −20 °C or −80 °C until use. Repeated freeze-thaw cycles were avoided.

**Figure 2:**
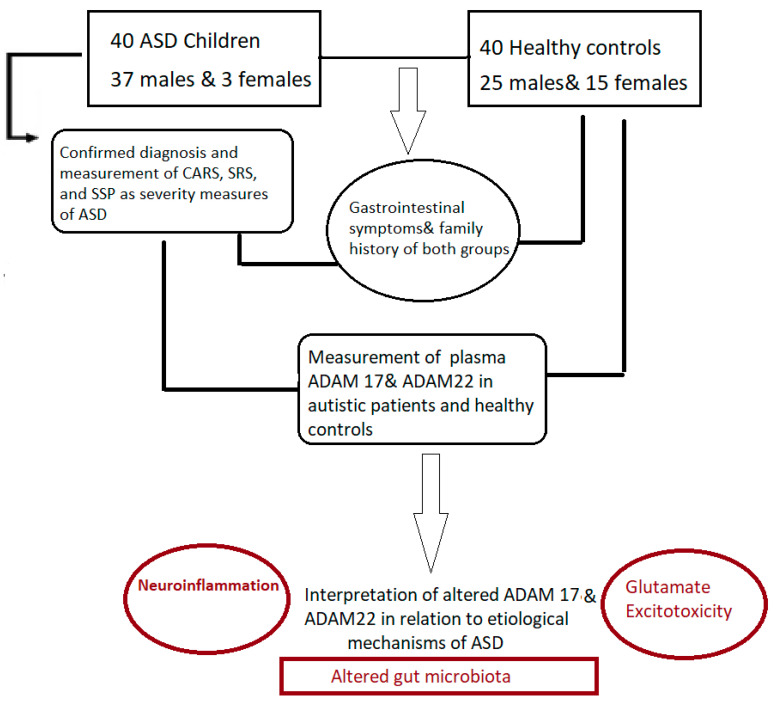
Illustration of the experimental design.

B Assay of ADAM17 and ADAM22 using ELISA technique:

EIAab Science INC, Wuhan’s ELISA kits were used to assay both metalloproteases. The microtiter plate for these kits has been pre-coated with either an antibody specific for ADAM 17 (Catalogue No. E1319h) or ADAM 22 (Catalogue No. E14575h). For each protein assay, the standards or samples were added to the microtiter plate wells first, followed by the addition of a biotin-conjugated polyclonal antibody preparation, specific for ADAM 17 or ADAM 22, and finally the addition of avidin conjugated to horseradish peroxidase (HRP) and incubated. To each well, a TMB substrate solution was then added. Only the ADAM17, or ADAM22 biotin-conjugated antibody, and enzyme-conjugated avidin-containing wells show a change of color which was measured at 450 nm wavelength using a spectrophotometer. The O.D. of the samples was then compared to the standard curve to determine the concentration of both proteins in the control and patient plasma samples. Detection limit was 78–5000 pg/mL for both. The summary of the experiment is illustrated in Figure 1 and Figure 2.

### 2.6. Statistical Analysis

The statistical analysis was conducted using software, and the results were presented as mean ± S.D. Independent Student’s *t*-tests were used for all statistical comparisons, with a *p* value < 0.05 being considered significant. When the Shapiro–Wilk’s test is negative and the data are not normally distributed, the Wilcoxon–Mann–Whitney test is typically utilized. The Spearman correlations test was used to calculate the association between the ADAM 17 and ADAM 22, CARS, SRS, SSP, and age. Positive or negative correlations were reported. The ROC curve was generated using the following parameters: (a) diagnostic sensitivity, demonstrating a proportion of true positive results in all patients with the analyzed pathology; (b) diagnostic specificity, demonstrating a proportion of true negative results in all healthy individuals without such pathology. AUC equal to 0.5 means no discrimination, 0.6–0.7 acceptable discrimination, 0.7–0.8 excellent discrimination, and AUC > 0.9 means outstanding discrimination.

## 3. Results

**Figure 3:**
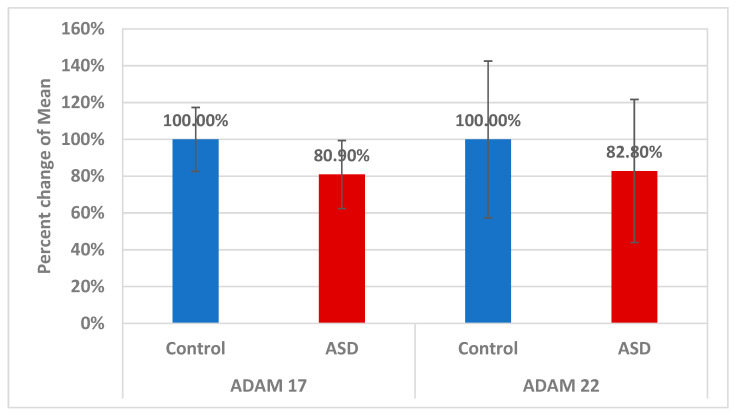
Shows the percentage difference between ASD patients and controls for ADAM17 and ADAM22. Both variable control levels were set at 100%, and it was estimated how much both decreased in people with ASD relative to control.

**Figure 4:**
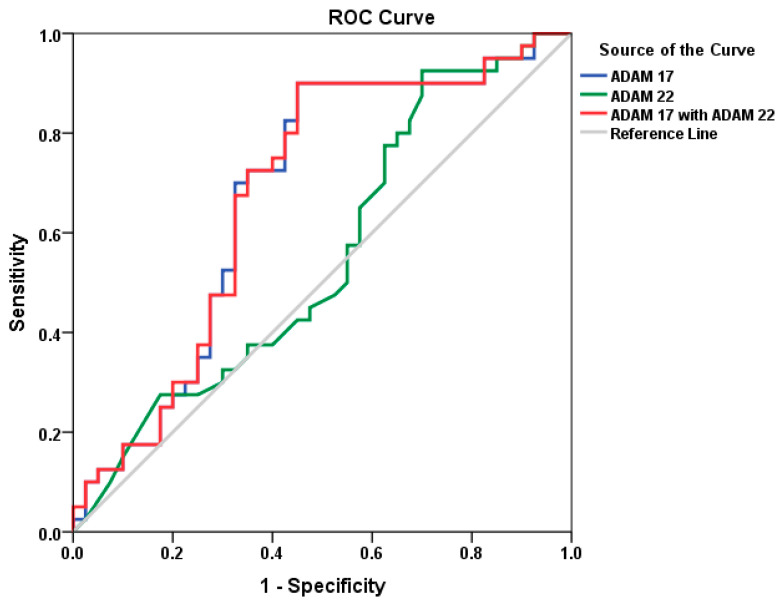
ROC of ADAM17 and ADAM22 of ASD patients group according to control group.

Table 1 compares ASD individuals to healthy controls in terms of parental age, gastrointestinal problems, family history, and brain diseases. While parental age was similar in both groups, gastrointestinal issues, specifically colic, constipation, diarrhea, itchy skin, and irritability, were significantly higher in ASD participants compared to controls. This may demonstrate the importance of the gut–brain axis as an etiological mechanism in ASD. ASD families had a family history of depression, bipolar disorder, and schizophrenia, but not control group families. The ratio of males to females was significantly higher among ASD participants compared to controls (*p* < 0.001) (Table 2). Table 3 demonstrates the mean ± S.D. of CARS, SRS, and SSP of the 40 participants with ASD. Values recorded 30.80 ± 3.75 in mild-moderate CARS against 43.05 ± 6.30 in severe patients. SRS as a measure of impaired social interaction recorded values of 65.86 ± 11.02 in mild-moderate patients and 130.91 ± 38.45 in ASD severe participants. SSP recorded 172.14 ± 15.33 in typical participants against 121.06 ± 22.88 in atypical ASD patients. Table 4 and Figure 3 present −19.1% significantly lower level of ADAM17 (*p* < 0.001) and a non-significant but still considerably lower level (−17.2%) of ADAM22 (*p* < 0.413) in ASD participants compared to controls. Table 5 demonstrates the effect of gender on ADAM17 and ADAM22 in ASD patients. It can be easily noticed that no significant differences were recorded in both variables. Table 6 demonstrates the Spearman correlations between the two measured variables and between each of them and CARS, SRS, and S.S. profile as three measures of ASD severity. While ADAM17 and ADAM 22 showed significant positive correlation between each other, ADAM 17 demonstrated significant negative correlation with age, and ADAM 22 presented significant negative correlation with CARS as measure of ASD cognitive dysfunction (*p* < 0.001). ADAM 17 and ADAM 22 showed significant differences between mild-moderate and severe autistic persons enrolled in the study for CARS as a measure of ASD severity (*p* < 0.041 and 0.05 respectively), but not in SRS or SSP (Table 7). Table 8 and Figure 4 show that ADAM17 and ADAM 22 have only fair predictive values of measure as ROC-AUCs of 0.677 and 0.553 respectively with a slightly higher AUC value as the combined ROC (AUC of 0.678).

Table 4 describes comparison between control group and patient group for ADAM17 and ADAM22 using Mann–Whitney Test (non-parametric data).

Table 7 describes comparison between mild to moderate group and severe for CARS and SRS, typical and atypical for SSP in ASD group using Mann–Whitney Test (non-parametric data).

## 4. Discussion

Transmembrane-TNF-(tmTNF-), the precursor of the pro-inflammatory soluble form of TNF-(sTNF-), is converted by ADAM17 as TNF-converting enzyme (TACE) [28,29,30]. ADAM17 releases sTNF by proteolytically cleaving tmTNF at the cell surface [31]. For TNF-induced inflammation in pathological circumstances and inflammatory diseases such as ASD, ADAM17 ectodomain shedding of sTNF- is essential.

ASD patients frequently experience gastrointestinal (GI) issues. Madra et al. [31] reported that children with ASD had more GI symptoms, including constipation and diarrhea, than their unaffected siblings. Two meta-analyses found comparable outcomes in children with ASD. Moreover, patients with ASD who have gastrointestinal issues may exhibit substantial behavioral manifestations such as anxiety, self-injury, and violence [32,33,34]. A growing body of research suggests that the gut microbiota is directly or indirectly linked to ASD symptoms, in part by impacting the immune system and metabolism [35,36]. In our study, the various GI dysbiosis symptoms as co-morbidity among autistic patients shown in Table 1 may be connected to the changed levels of ADAM17 and ADAM22 represented in Table 4. ADAM17 can modulate intestinal inflammation, barrier permeability, and inflammatory responses in the intestine by cleaving various cytokines, including TNF- and lymphotoxins [37,38]. The fact that blood levels of TNF-α are positively connected with the severity of ASD symptoms in the same participant cohort of our investigation [39], and more importantly, that TNF-α may be a critical cytokine biomarker in ASD, lends support to our suggested hypothesis. It is not yet clear if the abnormal level of TNF-α is due to pro-inflammatory conditions or to disturbed associated signaling that takes place throughout the pathogenesis of autism, such as the reported down regulation of ADAM17, which is as a TNF converting enzyme [40].

Our study showed a significantly lower level of ADAM17 in plasma of ASD participants compared to controls. It could be suggested that the reported lower level of ADAM17 in plasma would be accompanied by higher levels in the brains of participants with ASD. In the present study, the significant decrease of ADAM 17 (*p* < 0.001) and the non-significant but still considerable 20% lower levels of ADAM22 in the plasma of children with ASD compared to controls could be attributed to the disrupted blood–brain barrier (BBB) in patients with ASD [41], and to the significant increase in levels of GM1 autoantibodies in children with ASD compared to controls as previously reported by Mostafa and AL-ayadhi [42]. The anti-LGI1 IgG4 antibodies alter the binding of LGI1 with ADAM22 and decrease the post-synaptic levels of AMPA receptors together with increased internalization of the LGI1/ADAM complexes. Defects in the transport of ADAM17 and ADAM22 from the brain to blood might lead to an increased load of inflammatory markers and glutamate signaling toxicity within the brain of an individual with ASD. This hypothesis may be supported by earlier research by Ray et al. [23], which revealed a markedly higher amount of soluble ADAM17 in the brain tissue of patients with ASD. Gender has no effect on the level of ADAM17 and ADAM 22 in control or ASD participants (Table 5).

Based on the fact that a shared underlying mechanism of ASD has been identified as a developmental imbalance in brain circuit excitation/inhibition [43,44], the suggested increase of ADAM22 internalization in autistic patients compared to control subjects (Table 4) can be supported by considering the Danesi et al. report [45] that dysregulation of AMPAR trafficking is a major cause of cognitive and social impairments in ASD in males and females which did not show a significant difference in the level of both studied variables. AMPAR density was lower in postmortem brain samples from ASD patients, notably in the cerebellum and prefrontal cortex [46]. 

Table 6 shows the correlations between plasma ADAM17 and ADAM22 on the one hand and age, CARS, SRS, and SSP on the other. While ADAM17 correlates significantly with age, ADAM22 correlates significantly with CARS as a marker of ASD severity. The observed negative connection between plasma ADAM17 and age may be supported by the study of Ray et al. [23] in which brain ADAM17 assessed via Western blotting was found to be favorably linked with age in autistic people. This might support the suggested abnormalities in ADAM17 transit from the brain to the blood, which could result in an elevated load of TNF-α in the brain of ASD patients [47]. Excitingly, elevated levels of brain TNF-α enhance the neurotoxic impact of glutamate, resulting in a synergistic increase of neuronal cell death [48,49].

It may help to highlight the importance of glutamate excitotoxicity and neuroinflammation as two key contributors to the etiology of ASD given the strong negative correlation between plasma ADAM 22 and CARS as a measure of ASD severity as well as the highly significant positive correlations between ADAM17 as protein connected to inflammation and ADAM22 as marker directly associated with glutamate neurotoxicity. For CARS as a measure of ASD severity, ADAM 17 and ADAM 22 indicate substantial differences between mild-moderate and severe autistic people included in the study, but not for SRS or SSP (Table 7). This can help to suggest that CARS may reliably identify between children with mild-to-moderate (CARS average score of 33.85 ± 2.21) and severe autism (CARS average score of 42.85 ± 12.45), as well as autism and mental retardation, implying that it can be used to measure the severity of anxiety-like behavior and brain hyperactivity as certain domains of autism severity in Saudi children [50]. This suggestion is consistent with several studies that show complete agreement between the DSM-IV and CARS as a diagnostic tool and measure of severity. The absence of false negatives when using CARS to differentiate between those with autism and those with other developmental disorders, ascertain its validity as a perfect severity measure when compared to SRS and SSP. One of the study’s advantages was that only one expert evaluated individual CARS scores, minimizing the impact of subjective scoring disparities. As a result, there were potentially fewer confounding factors in the data under consideration. In addition, the psychologist who administered the test was unaware of the outcomes of the complementary measure.

Table 8 presents the area under the curves (AUCs), specificity. and sensitivity of ADAM17 and ADAM22 in the plasma of individuals with ASD. In an ideal ROC test, the curve passes through the top left angle, where the proportion of true positive tests is 100%, and the lower the bend of the curve, the lower the quality of the test. The AUC value is used to calculate the numerical value of the clinical relevance of the test. If the area under the ROC curve is 1, the test has 100% sensitivity (no false positives) and 100% specificity (no false positive results). It can be easily noticed that while ADAM17 presents an acceptable discrimination value (AUC = 0.677), ADAM22 demonstrates an AUC of only 0.553. Combining both variables recorded an AUC of 0.678 with satisfactory specificity and sensitivity. This may imply that these two metalloproteases when combined, appear to be responsible for initiating neuroinflammation and excitotoxicity, two critical pathways involved in ASD development. This suggestion could be supported by the significant differences between severe and mild-moderate ADAM17 (*p* < 0.041) and ADAM22 (*p* < 0.05) participants in regard to CARS as the most reliable severity score of ASD. This could be explained and supported by considering the in vitro study in which ADAM17 inhibition lowers constitutive and LPS-activated TNF-α, TNFR-1, and IL-6R production by microglial cells while increasing constitutive and LPS-activated microglial phagocytotic activity. On the other hand, SRS and SSP as a measure of social interaction impairment and sensory profile, respectively show no significant changes in both measured enzymes.

## 5. Conclusions

This study highlights neuroinflammation and glutamate excitotoxicity, as two ASD etiological mechanisms that may be exacerbated by the elevated expression and activity of brain ADAM17 and ADAM22. Reduced or inhibited levels of these targets may therefore prove exciting as therapeutic approaches for ASD. 

## Figures and Tables

**Table 1:** Parents age, GI symptoms, and family history of the ASD patients and control group.

Symptoms	Control (40)	Autism (40)
**Parents’ age**		
Father’s age during the conception of the child	40 ± 6	39.2 ± 5
Mother’s age during the conception of the child	32 ± 7	33.7 ± 6
**GI symptoms**		
Vomiting after eating	1 (2.5)	7 (18%)
Reflux bloting	3 (7.5)	15 (37.5%)
Diarrhea	2 (5)	8 (20%)
Constipation	5 (12.5)	17 (42.5%)
Colic	2 (5)	22 (55%)
Itchy skin	3 (7.5)	15 (37.5%)
Rash	3 (7.5)	6 (15%)
Irritability	2 (5)	25 (62.5%)
**Family History of:**		
Depression		7 (18%)
Bipolar		5 (12.5%)
Schizophrenia		3 (7.5%)
Family History of RA	2 (5)	4 (10%)
Family History of SLE	1 (0.4)	3 (7.5%)
Family History of MS	2 (5)	2 (5%)

**Table 2:** Percentage of male and female participants in ASD and control groups.

Parameters		Groups		*p* Value
		Control	ASD	
Sex	Male (n, %)	25, 62.50%	37, 92.50%	0.001
	Female (n, %)	15, 37.50%	3, 7.50%	
Age (Yrs) (Mean ± S.D.)		5.83 ± 3.22	6.43 ± 2.22	0.335

**Table 3:** Mean ± S.D. of CARS, SRS, and SSP as measures of severity of the ASD patients.

	Groups	N	Min.	Max.	Mean ± S.D.	Percent Change
CARS	Mild to Moderate	20	23.00	36.00	30.80 ± 3.75	100.00
	Severe	20	37.00	63.00	43.05 ± 6.30	139.77
SRS	Mild to Moderate	7	41.00	72.00	65.86 ± 11.02	100.00
	Severe	33	80.00	203.00	130.91 ± 38.45	198.78
S.S Profile	Typical	22	154.00	211.00	172.14 ± 15.33	100.00
	Atypical	18	76.00	152.00	121.06 ± 22.88	70.33

**Table 4:** Mean ± S.D. of ADAM17 and ADAM22 in autistic group compared to control group.

Genes	Groups	N	Min.	Max.	Mean ± S.D.	Percent Change	*p* Value
ADAM 17	Control	40	2.41	29.52	18.34 ± 6.37	100.00	0.006
	ASD	40	2.31	25.57	14.83 ± 5.49	80.90	
ADAM 22	Control	40	0.013	1.545	0.54 ± 0.46	100.00	0.413
	ASD	40	0.013	1.428	0.45 ± 0.35	82.80	

**Table 5:** Effect of gender on ADAM17 and ADAM22 in ASD patients.

Enzyme	Groups	N	Min.	Max.	Mean ± S.D.	Percent Change	*p* Value
ADAM 17	Male	37	2.31	25.57	14.64 ± 5.65	100.00	0.342
	Female	3	14.72	18.57	17.23 ± 2.17	117.73	
ADAM 22	Male	37	0.013	1.254	0.43 ± 0.33	100.00	0.440
	Female	3	0.168	1.428	0.72 ± 0.65	168.08	

**Table 6:** Correlations between the following parameters using Spearman correlation.

Parameters	R (Correlation Coefficient)	*p* Value	
ADAM 17 with ADAM 22	0.462 **	0.001	P ^a^
ADAM 17 with CARS	−0.258	0.108	N ^b^
ADAM 17 with SRS	−0.107	0.510	N ^b^
ADAM 17 with S.S Profile	0.062	0.704	P ^a^
ADAM 17 with Age	−0.381 **	0.001	N ^b^
ADAM 22 with CARS	−0.322 *	0.043	N ^b^
ADAM 22 with SRS	−0.209	0.196	N ^b^
ADAM 22 with S.S Profile	0.187	0.248	P ^a^
ADAM 22 with Age	−0.038	0.739	N ^b^

* Correlation is significant at the 0.05 level; ** Correlation is significant at the 0.01 level; ^a^ Positive Correlation; ^b^ Negative Correlation.

**Table 7:** Comparison between mild to moderate group and severe for CARS and SRS, typical and atypical for S.S profile in ASD group.

	Enzymes	Groups	N	Min.	Max.	Mean ± S.D.	Percent Change	*p* Value
CARS	ADAM 17	Mild to Moderate	20	6.77	25.57	16.59 ± 5.19	100.00	0.041
		Severe	20	2.31	19.54	13.07 ± 5.34	78.79	
	ADAM 22	Mild to Moderate	20	0.032	1.428	0.56 ± 0.41	100.00	0.050
		Severe	20	0.013	0.808	0.34 ± 0.25	60.88	
SRS	ADAM 17	Mild to Moderate	7	9.12	25.57	17.73 ± 5.81	100.00	0.182
		Severe	33	2.31	23.98	14.22 ± 5.31	80.20	
	ADAM 22	Mild to Moderate	7	0.032	1.254	0.52 ± 0.45	100.00	0.695
		Severe	33	0.013	1.428	0.43 ± 0.34	83.97	
SSP	ADAM 17	Typical	22	2.53	25.57	15.20 ± 5.74	100.00	0.786
		Atypical	18	2.31	23.98	14.39 ± 5.30	94.68	
	ADAM 22	Typical	22	0.032	1.428	0.51 ± 0.38	100.00	0.314
		Atypical	18	0.013	1.060	0.37 ± 0.31	71.94	

**Table 8:** ROC Results for ASD group according to control group as a reference group.

Enzymes	AUC	Cut-Off Value	Sensitivity %	Specificity %	*p* Value	95% CI
ADAM 17	0.677	19.994	90.0%	55.0%	0.006	0.555–0.799
ADAM 22	0.553	0.837	92.5%	30.0%	0.413	0.425–0.681
ADAM 17 with ADAM 22	0.678	----	90.0%	55.0%	0.006	0.556–0.799

## Data Availability

The datasets generated and analyzed during the current study are available from the corresponding author on reasonable request.
